# Weight loss interventions and obesity‐associated cancers in people with type 2 diabetes and overweight/obesity: A real‐world observational study

**DOI:** 10.1111/dom.70090

**Published:** 2025-09-03

**Authors:** Testimony Ipaye, Jonathan Goldney, Thomas J. Wilkinson, Francesco Zaccardi, Thomas Yates, Melanie J. Davies, Karen Brown, Dimitris Papamargaritis

**Affiliations:** ^1^ NIHR Leicester Biomedical Research Centre University Hospitals of Leicester NHS Trust Leicester UK; ^2^ Leicester Diabetes Centre Leicester General Hospital, University of Leicester Leicester UK; ^3^ Leicester Cancer Research Centre University of Leicester Leicester UK

**Keywords:** bariatric surgery, cancer, obesity, overweight, semaglutide, tirzepatide, TriNetX, type 2 diabetes, weight loss

## Abstract

**Aims:**

To evaluate whether weight‐loss interventions are associated with obesity‐associated cancers (OAC) in individuals with overweight/obesity and type 2 diabetes (T2D).

**Materials and Methods:**

This retrospective cohort study utilised the TriNetX federated research network. Three cohorts of adults with overweight/obesity and T2D, treated with either semaglutide, tirzepatide or bariatric surgery (BS) between June 2005 and June 2025, were propensity score matched (1:1) to cohorts treated with dipeptidyl peptidase‐4 inhibitors (DPP‐4i) using potential confounding factors. Using Cox regression analysis, we estimated hazard ratios (HRs) of composite and individual OAC: breast, colorectal, gallbladder, liver, multiple myeloma, oesophageal, ovarian, pancreatic, renal, gastric cardia, thyroid and uterine cancers.

**Results:**

In 64,178 matched pairs (mean follow‐up 911 days), semaglutide (vs. DPP‐4i) was associated with lower rates of composite OAC (HR: 0.88; 95% CI: 0.82–0.95), colorectal (0.80; 0.67–0.97), liver (0.75; 0.60–0.95) and pancreatic (0.76; 0.60–0.96) cancers. In 19,682 matched pairs (mean follow‐up 435 days), tirzepatide (vs. DPP‐4i) was associated with a non‐significant lower rate of composite OAC (0.84; 0.69–1.01) but a significant lower rate of ovarian cancer (0.31; 0.10–0.95). In 9642 matched pairs (mean follow‐up 1746 days), BS (vs. DPP‐4i) was associated with lower rates of composite OAC (0.85; 0.74–0.98), liver (0.56; 0.32–0.97) and uterine cancers (0.59; 0.38–0.90), and higher rates of gastric cardia cancer (10.54; 1.35–82.38) and oesophageal cancer (4.78; 1.04–21.87).

**Conclusions:**

Semaglutide and BS were associated with lower cancer rates in individuals with overweight/obesity and T2D, with non‐significant lower rates also observed with tirzepatide. These findings suggest weight‐loss interventions may contribute to cancer prevention in this population.

## 1 INTRODUCTION

Obesity is a chronic, progressive disease characterised by excessive fat accumulation^1^, ^2^ and rising global prevalence.^3^ It is a major risk factor for numerous complications, including type 2 diabetes (T2D), cardiovascular disease, cancer and death.^4^, ^5^ Both overweight/obesity and T2D are strongly associated with increased cancer incidence and cancer‐related deaths.^6^, ^7^ In 2012, an estimated 6% of global cancer cases were attributed to diabetes and obesity.^8^ Overweight/obesity has been causally linked to 13 types of cancers, including breast, colorectal, liver, pancreatic, ovarian and uterine cancers.^9^ As overweight/obesity has a major contribution to the development of T2D, it likely amplifies cancer risk and mortality through mechanisms such as insulin resistance, hyperglycaemia, and increased leptin levels.^10^ In Western countries, such as the UK, USA and Australia, cancer is now the leading cause of death among individuals with T2D.^11^, ^12^, ^13^

Overweight/obesity is a key modifiable risk factor for both T2D and cancer. Early evidence suggests that bariatric surgery (BS) is associated with a reduced incidence of obesity‐associated cancers (OAC) in people with obesity and T2D,^14^, ^15^ with the benefit being more for those achieving diabetes remission. Until recently, BS was the only treatment shown to achieve ≥15% sustained mean weight loss (WL) in this population, with a significant proportion of participants also reaching euglycaemia (mean HbA1c ≤6.5%).^16^, ^17^ However, the emergence of glucagon‐like peptide‐1 (GLP‐1) receptor agonist (RA)‐based therapies has provided effective pharmacological treatments for treating overweight/obesity and T2D.

Semaglutide 2.4 mg, the approved dose for overweight/obesity management,^18^ is the most effective GLP‐1 receptor monoagonist, achieving 9.6% WL in individuals with T2D compared to 7% with the lower dose of 1 mg (which is approved for T2D management) and 3.4% with placebo.^19^ It also markedly improves glycaemic control, with 67.5% of participants reaching HbA1c ≤6.5%, versus 60.1% with semaglutide 1 mg and 15.5% with placebo. In the SURMOUNT 2 trial,^20^ tirzepatide 15 mg—a dual agonist acting on both GLP‐1 and glucose‐dependent insulinotropic polypeptide (GIP) receptors—led to 14.7% mean WL in people with T2D and overweight/obesity vs. 3.2% WL with placebo after 72 weeks of treatment; moreover, 79% of participants achieved HbA1c ≤6.5% with tirzepatide 15 mg compared to 20% with placebo.

In addition to achieving significant WL and euglycaemia, studies in preclinical models with obesity suggest that semaglutide and tirzepatide may also inhibit tumour development.^21^, ^22^, ^23^, ^24^, ^25^ The anti‐cancer effects of semaglutide and tirzepatide may be related to both weight‐dependent and weight‐independent pathways; for example, semaglutide is associated with a reduction in inflammation and oxidative stress.^26^, ^27^

In the present study, we adopted an active‐comparator, drug‐initiator, cohort design, using dipeptidyl peptidase‐4 inhibitors (DPP‐4i) as a comparator, in the large electronic health records network, TriNetX, to investigate the association of semaglutide, tirzepatide and BS with the rate of OAC in those with overweight/obesity and T2D. DPP‐4i were selected as the comparator group because, like semaglutide and tirzepatide, they are incretin‐based therapies,^28^ are commonly prescribed second‐line glucose‐lowering medications,^29^ and have shown a neutral association with cancer risk, including in a large meta‐analysis of randomised controlled trials (RCTs).^30^

## 2 MATERIALS AND METHODS

This study follows the Reporting of Studies Conducted using Observational Routinely‐collected Health Data (RECORD) guidelines (checklist reported in the Supporting Information).

### 2.1 Data source

This retrospective cohort study utilised data from the US Collaborative Network within the TriNetX federated research database.^31^ The US Collaborative Network consists of de‐identified pseudo‐anonymised electronic health record data, contributed to by multiple healthcare organisations (HCOs) across the US. Data from various HCOs are harmonised into a central database, including demographic data, diagnostic codes, medication, laboratory results and procedures.^31^ Data are available from the network to be used in real time for research purposes, utilising an in‐built analysis platform, which returns aggregate‐level results to the researcher. All data collection, processing and transmission were performed in accordance with all Data Protection laws applicable to the contributing HCOs, including the US Health Insurance Portability and Accountability Act. Ethical approval and consent were not required due to the anonymisation process and utilisation of routinely collected data.

### 2.2 Study population

We compared outcomes across four cohorts. All cohorts included people with T2D with the first‐ever prescription of (a) semaglutide; (b) tirzepatide; (c) individuals undergoing a BS procedure (defined as any code listed in Table S1); (d) any dipeptidyl peptidase‐4 inhibitor (DPP‐4i; the control cohort) at any time during the 20‐year period prior to the date of analysis (12th June 2025). The index date was defined as the first prescription of semaglutide/tirzepatide/DPP‐4i or the procedural code for BS. Prior to the index date, individuals were excluded if they had previous OAC, type 1 diabetes, body mass index (BMI) or HbA1c not recorded in the previous 12 months (so they were available for cohort matching); BMI <25 kg/m^2^ was recorded in the last 12 months; or there was previous use of tirzepatide, any GLP‐1 RA, DPP‐4i, or previous BS.

### 2.3 Outcomes

We investigated the rate of the following OACs: breast, colorectal, gallbladder, liver, multiple myeloma, oesophageal, ovarian, pancreatic, renal, gastric cardia, thyroid and uterine cancers, both individually and as a composite outcome (the first occurrence of any OAC).^9^ The clinical codes used to identify these outcomes are presented in Table S2.

### 2.4 Confounding factors

From HCO records harmonised on the TriNetX platform, potential confounding factors were identified up to 12 months prior to the index date (initiation of semaglutide/tirzepatide/DPP‐4i or date of BS). Confounding factors included demographics: age at index, sex (male/female/unknown), race (American Indian or Alaska native/Asian/Black or African American/Native Hawaiian or Other Pacific Islander/White/other race/unknown race); ethnicity (Hispanic or Latino/Not Hispanic or Latino/Unknown ethnicity); anthropometrics (BMI; categorised in 5 kg/m^2^ increments between 25 and 55 kg/m^2^, with categories for values below 25 kg/m^2^ and above 55 kg/m^2^); biochemistry (HbA1c; categorised in 1% increments between 6.5% and 11.5%, with categories for values below 6.5% and above 11.5%); past medical history (each yes/no): hypertensive diseases, ischaemic heart diseases, kidney complications, neurological complications, ophthalmic complications, cerebrovascular diseases, peripheral vascular disease, atherosclerosis, nicotine dependence, alcohol‐related disorders, personal history of nicotine dependence; social factors (persons with potential health hazards related to socioeconomic and psychosocial circumstances [yes/no]); glucose‐lowering therapies (each yes/no): metformin, insulin, sodium‐glucose co‐transporter 2 inhibitors, sulfonylureas. All clinical codes used to identify relevant confounding factors are reported in Table S3.

### 2.5 Statistical analysis

We investigated the rates of all major OAC in the semaglutide, tirzepatide and BS cohorts compared to matched DPP‐4i cohorts. Baseline characteristics for each exposed cohort (semaglutide, tirzepatide, BS) as compared to the control cohort (DPP‐4i) were calculated separately, both before and after propensity score matching, with standardised mean differences reported for all matched variables. Continuous variables were reported as mean and standard deviation (SD), and categorical variables as number and percentage. Propensity score matching utilised 1:1 “greedy nearest neighbour” with a calliper width of 0.1 SD, including the previously mentioned confounding variables.

The time axis started from the index date (initiation of treatment) at any HCO in the TriNetX network, with data censored at the occurrence of an outcome, or at the most recent visit to an HCO within the TriNetX network before the date of analysis (12th June 2025). The Cox proportional‐hazards model was used to assess the hazard ratios (HRs) of composite cancer and each individual OAC (breast, colorectal, gallbladder, liver, multiple myeloma, oesophageal, ovarian, pancreatic, renal, gastric cardia, thyroid and uterine), comparing each exposure cohort (semaglutide, tirzepatide, BS) to the corresponding matched DPP‐4i cohort. Analyses were repeated in women and men separately. Additionally, a sensitivity analysis was undertaken excluding individuals diagnosed with cancer within 6 months of initiation of treatment due to a theoretical risk of reverse causality (i.e., early signs and symptoms of cancer influencing choice of glucose‐lowering therapeutic agent), ensuring that the exposure preceded the outcome and limited any potential detection bias of cancer related to initiation of therapies.

All HRs were reported with 95% confidence intervals (CIs), with an upper bound 95% CI <1, or a lower bound 95% CI >1, considered statistically significant.

Analyses were performed on the TriNetX platform, which utilises R's survival package, version 3.2‐3, to undertake Cox regression modelling. To safeguard protected health information, when presenting results, counts of <10 were not shown. Figures were created in Stata v18.0.

## 3 RESULTS

### 3.1 Baseline characteristics

Table 1 shows the number of individuals identified in the semaglutide, tirzepatide, BS cohort after matching. There were 64,178 matched pairs for the semaglutide vs. DPP‐4i analysis (mean age at index date: 59.9 vs. 60.3 years; 48.1% vs. 47.7% women, respectively), 19,682 matched pairs for tirzepatide vs. DPP‐4i comparison (mean age at index date: 58.8 vs. 59.4 years; 49.6% vs. 48.7% women, respectively) and 9,642 matched pairs for the BS vs. DPP‐4i analysis (mean age at index date: 51.2 vs. 51.1 years; 67.7% vs. 67.4% women, respectively). In general, race, ethnicity, BMI, HbA1c, past medical history and use of glucose‐lowering therapies were well matched in all analyses. Baseline characteristics before propensity score matching are reported in Table S4.

**TABLE 1:** Baseline characteristics after propensity score matching. Data are presented as number (%) for categorical variables and mean (SD) for continuous variables.

	Semaglutide versus DPP‐4i			Tirzepatide versus DPP‐4i			Bariatric surgery versus DPP‐4i		
	Exposure	Comparator (DPP‐4i)	SMD	Exposure	Comparator (DPP‐4i)	SMD	Exposure	Comparator (DPP‐4i)	SMD
People	64 178	64 178	—	19 682	19 682	—	9642	9642	—
Current age	63.0 (11.6)	63.3 (12.9)	0.026	60.4 (11.6)	60.8 (13.3)	0.036	57.8 (12.0)	57.7 (13.8)	0.007
Age at index	59.9 (11.8)	60.3 (13.3)	0.028	58.8 (11.8)	59.4 (13.6)	0.041	51.2 (11.3)	51.1 (13.4)	0.011
Sex									
Female	30 888 (48.1)	30 663 (47.7)	0.007	9780 (49.6)	9587 (48.7)	0.020	6529 (67.7)	6499 (67.4)	0.007
Male	31 143 (48.5)	31 353 (48.8)	0.007	9391 (47.7)	9542 (48.4)	0.015	2806 (29.1)	2861 (29.6)	0.013
Unknown	2147 (3.34)	2162 (3.36)	0.001	511 (2.59)	553 (2.81)	0.013	307 (3.18)	282 (2.92)	0.015
Race									
American Indian or Alaska Native	380 (0.59)	331 (0.51)	0.010	109 (0.55)	96 (0.48)	0.009	37 (0.38)	38 (0.39)	0.002
Asian	3146 (4.90)	3186 (4.96)	0.003	754 (3.83)	792 (4.02)	0.010	114 (1.18)	129 (1.33)	0.014
Black or African American	11 985 (18.6)	12 114 (18.8)	0.005	3433 (17.4)	3427 (17.4)	<0.001	2140 (22.1)	2100 (21.7)	0.010
Native Hawaiian or Other Pacific Islander	685 (1.06)	636 (0.99)	0.008	134 (0.68)	129 (0.65)	0.003	54 (0.56)	74 (0.76)	0.026
White	39 892 (62.1)	39 722 (61.8)	0.005	12 893 (65.5)	12 810 (65.0)	0.009	5990 (62.1)	6001 (62.2)	0.002
Other race	2669 (4.15)	2696 (4.20)	0.002	849 (4.31)	869 (4.41)	0.005	429 (4.44)	437 (4.53)	0.004
Unknown race	5421 (8.44)	5493 (8.55)	0.004	1510 (7.67)	1559 (7.92)	0.009	878 (9.10)	863 (8.95)	0.005
Ethnicity									
Hispanic or Latino	6408 (9.98)	6486 (10.1)	0.004	1979 (10.0)	2008 (10.2)	0.005	1249 (12.9)	1252 (12.9)	<0.001
Not Hispanic or Latino	44 404 (69.1)	44 313 (69.0)	0.003	13 184 (66.9)	13 122 (66.6)	0.007	6821 (70.7)	6883 (71.3)	0.014
Unknown ethnicity	13 366 (20.8)	13 379 (20.8)	<0.001	4519 (22.9)	4552 (23.1)	0.004	1572 (16.3)	1507 (15.6)	0.018
BMI kg/m^2^	35.1 (7.33)	34.3 (7.29)	0.115	36.0 (7.39)	34.8 (7.27)	0.176	43.3 (8.26)	42.8 (8.17)	0.057
BMI categories (kg/m^2^)									
25–30	21 262 (33.1)	21 883 (34.0)	0.020	5344 (27.1)	5739 (29.1)	0.045	440 (4.56)	489 (5.07)	0.024
30–35	25 665 (39.9)	25 534 (39.7)	0.004	7849 (39.8)	7948 (40.3)	0.010	1088 (11.2)	1118 (11.5)	0.010
35–40	18 492 (28.8)	18 153 (28.2)	0.012	6172 (31.3)	6010 (30.5)	0.018	3498 (36.2)	3494 (36.2)	<0.001
40–45	10 457 (16.2)	10 215 (15.9)	0.010	3543 (18.0)	3364 (17.0)	0.024	4041 (41.9)	4068 (42.1)	0.006
45–50	5319 (8.28)	5142 (8.01)	0.010	1765 (8.96)	1645 (8.35)	0.022	2921 (30.2)	3012 (31.2)	0.020
50–55	2493 (3.88)	2419 (3.76)	0.006	873 (4.43)	799 (4.06)	0.019	1720 (17.8)	1778 (18.4)	0.016
>55	1625 (2.53)	1590 (2.47)	0.003	574 (2.91)	527 (2.67)	0.014	1332 (13.8)	1349 (13.9)	0.005
HbA1c %	8.15 (1.94)	8.24 (1.97)	0.043	7.90 (1.95)	8.06 (1.95)	0.078	6.93 (1.40)	7.35 (1.65)	0.276
HbA1c categories (%)									
<6.5	16 279 (25.3)	16 109 (25.1)	0.006	6103 (31.0)	5991 (30.4)	0.012	4800 (49.7)	4767 (49.4)	0.007
6.5–7.5	26 207 (40.8)	26 310 (40.9)	0.003	8380 (42.5)	8395 (42.6)	0.002	4033 (41.8)	4008 (41.5)	0.005
7.5–8.5	20 489 (31.9)	20 497 (31.9)	<0.001	5437 (27.6)	5566 (28.2)	0.015	2057 (21.3)	2131 (22.1)	0.019
8.5–9.5	12 705 (19.7)	12 702 (19.7)	<0.001	3176 (16.1)	3226 (16.3)	0.007	1042 (10.8)	1127 (11.6)	0.028
9.5–10.5	7882 (12.2)	7774 (12.1)	0.005	2036 (10.3)	2083 (10.5)	0.008	557 (5.77)	593 (6.15)	0.016
10.5–11.5	5271 (8.21)	5226 (8.14)	0.003	1369 (6.95)	1371 (6.96)	<0.001	286 (2.96)	307 (3.18)	0.013
>11.5	6608 (10.2)	6628 (10.3)	0.001	1837 (9.33)	1832 (9.30)	<0.001	301 (3.12)	305 (3.16)	0.002
Past medical history									
Hypertensive diseases	48 899 (76.1)	49 229 (76.7)	0.012	14 390 (73.1)	14 561 (73.9)	0.020	7523 (78.0)	7521 (78.0)	<0.001
Ischemic heart diseases	12 751 (19.8)	12 925 (20.1)	0.007	3341 (16.9)	3455 (17.5)	0.015	1388 (14.3)	1487 (15.4)	0.029
Type 2 diabetes mellitus with kidney complications	10 890 (16.9)	11 265 (17.5)	0.015	2872 (14.5)	3029 (15.3)	0.022	888 (9.21)	990 (10.2)	0.036
Type 2 diabetes mellitus with neurological complications	8841 (13.7)	9085 (14.1)	0.011	2433 (12.3)	2526 (12.8)	0.014	875 (9.07)	908 (9.41)	0.012
Nicotine dependence	6602 (10.2)	6649 (10.3)	0.002	1843 (9.36)	1875 (9.52)	0.006	897 (9.30)	900 (9.33)	0.001
Cerebrovascular diseases	4985 (7.76)	5147 (8.02)	0.009	1223 (6.21)	1297 (6.59)	0.015	362 (3.75)	351 (3.64)	0.006
Type 2 diabetes mellitus with ophthalmic complications	3142 (4.89)	3202 (4.98)	0.004	890 (4.52)	897 (4.55)	0.002	265 (2.74)	272 (2.82)	0.004
Atherosclerosis	2835 (4.41)	2967 (4.62)	0.010	789 (4.00)	809 (4.11)	0.005	183 (1.89)	170 (1.76)	0.010
Personal history of nicotine dependence	8173 (12.7)	8385 (13.0)	0.010	2315 (11.7)	2410 (12.2)	0.015	1807 (18.7)	1919 (19.9)	0.029
Peripheral vascular disease, unspecified	2528 (3.93)	2596 (4.04)	0.005	643 (3.26)	686 (3.48)	0.012	195 (2.02)	189 (1.96)	0.004
Alcohol related disorders	1303 (2.03)	1329 (2.07)	0.003	390 (1.98)	422 (2.14)	0.011	202 (2.09)	216 (2.24)	0.010
Glucose‐lowering therapies									
Metformin	37 104 (57.8)	37 032 (57.7)	0.002	10 297 (52.3)	10 319 (52.4)	0.002	3951 (40.9)	3955 (41.0)	<0.001
Insulin	20 516 (31.9)	24 660 (38.4)	0.136	5873 (29.8)	6126 (31.1)	0.028	5051 (52.3)	5265 (54.6)	0.045
SGLT2i	12 807 (19.9)	12 693 (19.7)	0.004	4176 (21.2)	4388 (22.2)	0.026	380 (3.94)	373 (3.86)	0.004
Sulfonylureas	13 099 (20.4)	13 241 (20.6)	0.005	2977 (15.1)	2973 (15.1)	<0.001	973 (10.0)	1063 (11.0)	0.030
Persons with potential health hazards related to socioeconomic and psychosocial circumstances	2020 (3.14)	2024 (3.15)	<0.001	721 (3.66)	786 (3.99)	0.017	265 (2.74)	310 (3.21)	0.012

Abbreviations: BMI, body mass index; DBP, diastolic blood pressure; eGFR, estimated glomerular filtration rate; SBP, systolic blood pressure; SGLT2i, sodium‐glucose cotransporter‐2 inhibitors; SMD, standardised mean difference.

### 3.2 Cancer outcomes

**FIGURE 1:**
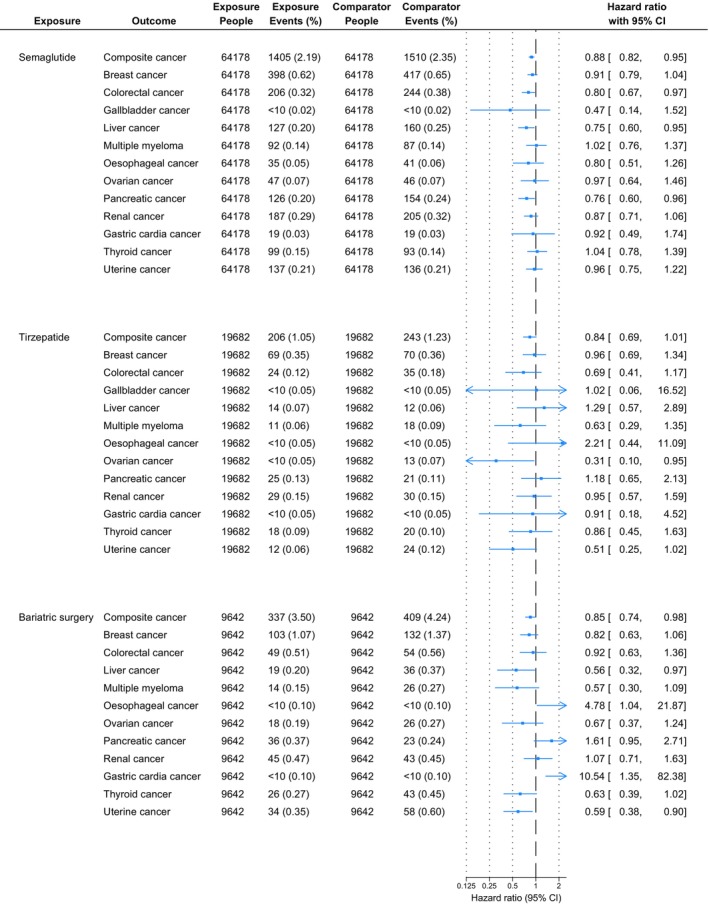
Hazard ratio of obesity‐associated cancers in individuals with type 2 diabetes and overweight/obesity treated with semaglutide, tirzepatide, or bariatric surgery, as compared to DPP‐4 inhibitors. The hazard ratio of gallbladder cancer could not be calculated for bariatric surgery cohorts as compared to the DPP‐4 inhibitor cohort due to no events occurring in one or more cohorts. CI, confidence interval.

The mean (SD) follow‐up time was 911 (557) days for the semaglutide group and 864 (666) days for the DPP‐4i group in the semaglutide versus DPP‐4i analysis (Table S5). In the tirzepatide versus DPP‐4i analysis, follow‐up was 435 (279) days for tirzepatide and 439 (389) days for DPP‐4i. For the BS versus DPP‐4i analysis, follow‐up times were 1746 (1395) days and 1823 (1358) days, respectively. Figure 1 shows the relative rates of OAC with semaglutide, tirzepatide and BS versus DPP‐4i in individuals with T2D and obesity.

#### 3.2.1 Semaglutide versus DPP‐4i

Semaglutide was associated with a significantly lower rate of composite OAC, with a HR of 0.88 (95% CI: 0.82–0.95), compared to DPP‐4i, with 1405 events for semaglutide and 1510 events for DPP‐4i. For individual OAC, semaglutide was associated with significantly lower rates compared to DPP‐4i of colorectal cancer (0.80; 0.67–0.97) (206 events, semaglutide; 244 events, DPP‐4i), liver cancer (0.75; 0.60–0.95) (127 events, semaglutide; 160 events, DPP‐4i) and pancreatic cancer (0.76; 0.60–0.96) (126 events, semaglutide; 154 events, DPP‐4i), with no statistically significant difference in the relative hazards of any other cancers.

#### 3.2.2 Tirzepatide versus DPP‐4i

Tirzepatide was associated with a lower rate of composite OAC compared to DPP‐4i (HR: 0.84; 95% CI: 0.69–1.01) with 206 events for tirzepatide and 243 for DPP‐4i, although this was not statistically significant. For individual OACs, tirzepatide was associated with a lower rate of ovarian cancer as compared to DPP‐4i (0.31; 0.10–0.95) (<10 events, tirzepatide; 13 events, DPP‐4i), but was not associated with significantly different rates of other individual OACs.

#### 3.2.3 BS versus DPP‐4i

BS was associated with a significantly lower rate of composite OAC vs. DPP‐4i (HR: 0.85; 95% CI: 0.74–0.98) with 337 events for BS and 409 events for DPP‐4i. For individual OAC, BS was associated with statistically significantly lower rates compared to DPP‐4i for liver cancer (0.56; 0.32–0.97) (19 events, BS; 36 events, DPP‐4i) and uterine cancer (0.59; 0.38–0.90) (34 events, BS; 58 events, DPP‐4i) and an increased rate of gastric cardia cancer (10.54; 1.35–82.38) [<10 events each] and oesophageal cancer (4.78; 1.04–21.87) (<10 events each); there was no statistically significant difference in the rates of other OACs.

### 3.3 Sex stratification

**FIGURE 2:**
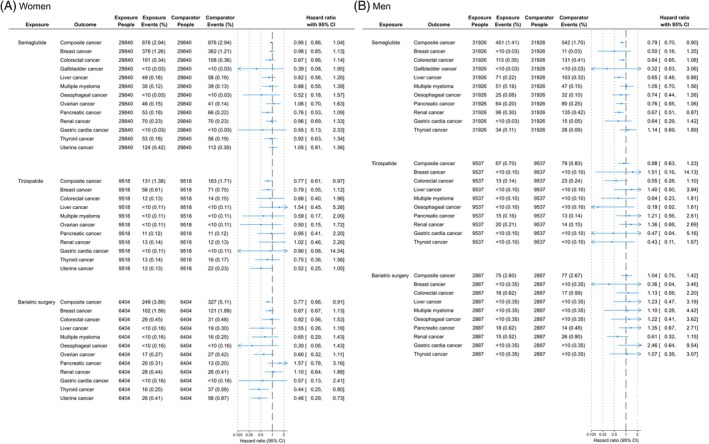
Sex stratified analysis of individuals with type 2 diabetes and overweight/obesity treated with semaglutide, tirzepatide, or bariatric surgery, as compared to DPP‐4 inhibitors. The hazard ratio of individual cancers could not be calculated in all analyses due to no events occurring in one or more cohorts; this includes: gallbladder cancer (tirzepatide and bariatric surgery; both men and women), oesophageal cancer (tirzepatide; women), uterine (analyses in men), and ovarian cancer (analyses in men). CI, confidence interval.

When analyses were stratified by sex (Figure 2), semaglutide was associated with a lower rate of composite OAC as compared to DPP‐4i in men only (HR: 0.79; 95% CI: 0.70–0.90), being not significantly lower in women (0.95; 0.86–1.04). For tirzepatide this pattern was reversed, with women having a lower rate of OAC with tirzepatide vs. DPP‐4i (0.77; 0.61–0.97), whereas the lower rate was not statistically significant in men (0.88; 0.63–1.23). Similarly, BS was associated with a low rate of OAC vs. DPP‐4i in women (0.77; 0.66–0.91) but not in men (1.04; 0.75–1.42). By sex, for individual cancers, across all exposures, the only statistically significant differences in rates were observed in women who underwent BS vs. DPP‐4i with lower rates of thyroid (0.44; 0.25–0.80) and uterine cancer (0.46; 0.29–0.73).

### 3.4 Sensitivity analysis

In the sensitivity analysis (Figure S6), excluding individuals that developed OAC within the first 6 months of follow‐up, semaglutide was no longer associated with a lower rate of OAC versus DPP‐4i (HR: 1.00; 95% CI: 0.92–1.09); tirzepatide was associated with a similar lower rate of OAC; however, this remained not significant (0.81; 0.62–1.05); and BS remained associated with a significant lower rate of OAC versus DPP‐4i (0.82; 0.70–0.96). For individual cancers, only tirzepatide (0.32; 0.11–0.87) and BS (0.53; 0.34–0.81) were significantly associated with lower rates of uterine cancer, whilst BS was also significantly associated with lower rates of liver cancer versus DPP‐4i (0.49; 0.26–0.91). No other significant differences were observed for other exposures or other individual OACs.

## 4 DISCUSSION

### 4.1 Summary of findings

To the best of our knowledge, this is the first study to comprehensively assess the rate of OAC across multiple weight‐lowering interventions in individuals with T2D and overweight/obesity, and the first study to investigate the OAC rate with tirzepatide use in a real‐world population. We observed that, in individuals with T2D and overweight/obesity, in the primary analysis, semaglutide and BS were associated with a lower rate of composite OAC outcome compared to DPP‐4i use, 12% and 15% lower rates, respectively. A trend for a lower rate of composite OAC compared to DPP‐4i (16% lower) was also seen with tirzepatide, which did not meet the threshold for statistical significance. As compared to DPP‐4i, semaglutide was also associated with a significantly lower rate of colorectal, liver and pancreatic cancer; tirzepatide was associated with a lower rate of ovarian cancer; and BS was associated with a lower rate of liver and uterine cancer, although the rates of gastric cardia and oesophageal cancer were higher.

Whilst the findings from the primary analysis show a clear pattern of reduced OAC rates with weight‐lowering interventions, the lower rate of the composite OAC outcome with semaglutide versus DPP‐4i should be interpreted with caution, as there was no difference in OAC rates between semaglutide and DPP‐4i in the sensitivity analysis excluding individuals who developed cancer within the first 6 months of follow‐up. Although the reason remains unclear, there may be two possible explanations why rates were reduced with semaglutide in the main analysis, but not after excluding individuals that developed cancer within the first 6 months. Firstly, in real‐world studies, semaglutide has been associated with high rates of treatment discontinuation,^32^, ^33^ with 46.5% of individuals with T2D discontinuing GLP‐1 RA treatment within 1 year of initiation in a large US study of electronic medical records.^33^ Treatment discontinuation, in turn, is associated with weight‐regain.^34^ Semaglutide may exert anti‐tumour effects that are not solely dependent on WL, potentially including reductions in systemic inflammation and direct actions on tumour cell biology.^35^, ^36^ Early treatment discontinuation may interrupt these weight‐independent mechanisms, potentially permitting tumour progression to resume. Furthermore, discontinuation is often accompanied by weight regain, which may further diminish any protective effect. These observations suggest that both the WL and any associated reduction in OAC risk may be transient if therapy is not maintained. However, in the case of tirzepatide, although discontinuation rates are similarly high, the relative rates of cancer remained largely consistent between the primary and sensitivity analyses— though they did not reach statistical significance.^32^ The second possible explanation for the discrepancy in findings between the primary and sensitivity analyses could be that the results are affected by reverse causality. In particular, physicians may be less likely to initiate semaglutide as compared to DPP‐4i in individuals presenting with the early signs and symptoms of undiagnosed cancer, particularly because of the more common and severe adverse effect profile associated with semaglutide as compared to DPP‐4i, and because weight loss itself is a common sign of cancer, and therefore a weight‐neutral therapy may be preferentially chosen. This may lead to a falsely high rate of OAC in the group initiating DPP‐4i within the first 6 months, leading to a reduction in HR comparing semaglutide to DPP‐4i. Despite this, if reverse causality was the primary driver of the lower rate of OAC with semaglutide in the primary analysis, the lack of association would be similarly expected in the sensitivity analysis with tirzepatide; however, the HR was largely unchanged. Additional prospective evidence with greater statistical power is required to clarify further; however, for now, the rate reduction with semaglutide should be interpreted with caution.

When results were stratified by sex, semaglutide was associated with a significantly lower rate of OAC vs. DPP‐4i in men but not women. This is surprising, as WL associated with semaglutide has been shown to be greater in women as compared to men; for example, in the SELECT trial, the estimated treatment difference (ETD) with semaglutide vs. placebo was −11.11% (95% CI: −11.56, −10.66) in women vs. −7.50% (−7378, −7.23) in men at week 104.^37^ Whilst the reason for this difference remains unclear, OACs represent a heterogeneous group of cancers, each with separate aetiological processes and it may be that semaglutide is not as effective in lowering breast and uterine cancers compared to other OACs which are the most common OACs in women. On the other hand, we observed that tirzepatide was associated with a significant rate reduction in OAC versus DPP‐4i in women but not men: 23% versus 12% lower rate, respectively. Given that fewer events occurred within the tirzepatide as compared to the semaglutide analysis, it remains unclear if this represents a true sex difference or reflects a lack of statistical power in the men subgroup. BS was also associated with a significantly lower rate of OAC versus DPP‐4i in women but not men. Our findings reflect those of a secondary analysis of the prospective controlled SOS study of BS in individuals with T2D and obesity, where a significant reduction in cancer was associated with BS versus control in women (adjusted HR: 0.58; 95% CI: 0.38–0.90) but not men (0.79; 0.46–1.38), although a difference in treatment effect by sex was not significant (interaction *p* = 0.63).^38^ Whilst other studies have demonstrated lower cancer with BS in women but not men,^39^ due to fewer events and less men vs. women having BS, both our study and the SOS study likely lacked power to detect a reduction in OAC in men and therefore it remains unknown whether there may be true sex differences in cancer prevention with BS.

### 4.2 Findings in the context of previous literature

#### 4.2.1 Semaglutide

Although our findings regarding rate reductions in composite OAC with semaglutide should be interpreted with caution due to the lack of association in the sensitivity analysis (exclusion of individuals that developed cancer within 6 months), our findings of reductions in OAC, colorectal, liver and pancreatic cancer are broadly consistent with the previous literature investigating the effect of GLP‐1 RAs on cancer incidence. Although early observational studies had raised concerns regarding a potential increased risk of pancreatic and thyroid cancers with GLP‐1 RAs,^40^, ^41^, ^42^ more recent analyses have not supported this association.^43^, ^44^ A meta‐analysis by Nagendra et al., with data from 37 randomised controlled trials and 19 real‐world studies, found no increased risk of pancreatic cancer (odds ratio [OR] 0.25 [95% CI: 0.03–2.24]), thyroid cancer (2.04; 0.33–12.61), or other neoplasms (0.95; 0.62–1.45) in participants using semaglutide,^45^ albeit there was a large degree of statistical uncertainty. A TriNetX study by Arunkumar et al. compared pancreatic cancer rates in individuals using GLP‐1 RAs versus those on metformin, reporting a 53% rate reduction (HR = 0.47; 95% CI, 0.42–0.52), which aligns with our results.^46^ Moreover, Wang et al. found in another TriNetX study a 23% lower rate (HR = 0.77; 95% CI, 0.59–1.00) in colorectal cancer with GLP‐1 RAs versus DPP‐4i in drug‐naïve people with T2D and overweight or obesity, an effect size similar to our findings for semaglutide. In a further study by Wang et al., GLP‐1 RAs, compared to insulin, were associated with a lower rate of 10 individual OACs, including colorectal, liver and pancreatic cancer.^47^ However, GLP‐1 RAs were not significantly associated with a lower rate of colorectal, pancreatic or liver cancer compared to metformin. It is important to note the differences in our results compared to Wang et al. may be attributed to the use of different comparators and a greater number of variables in our propensity score matching analysis. Notably, our adjustment may be more robust, as Wang et al. accounted for BMI but did not adjust for HbA1c, an objective marker of glycaemic control.

The clinical trial and real world data from humans is supported by emerging preclinical studies in rodents suggesting that semaglutide has potential benefits against the development and/or treatment of several individual OACs, including hepatocellular carcinoma related to metabolic‐dysfunction associated steatohepatitis,^35^ endometrial and breast cancer.^48^, ^49^ Such models will be useful to delineate mechanisms of action and in particular ascertain whether there are any WL‐independent effects of semaglutide that contribute to cancer preventive activity.

#### 4.2.2 Tirzepatide

Whilst we did observe a significantly lower rate of OAC in women, and a lower rate of ovarian cancer in our primary analysis, the 16% lower rate of composite OAC with tirzepatide versus DPP‐4i in the primary analysis narrowly missed the threshold for statistical significance. Despite this, other evidence suggests that tirzepatide may have potent anti‐cancer effects. Firstly, tirzepatide results in greater WL and HbA1c reduction than other GLP‐1 based therapies, even at the dose of 5 mg once weekly (the lowest approved dose for obesity and T2D management).^50^ Additionally, the dual GLP‐1 and GIP receptor agonism with tirzepatide may provide unique metabolic and immunomodulatory benefits,^51^ potentially enhancing its protective effect against OAC.

Studies consistently show a trend towards lower rates of cancer with tirzepatide, although seemingly also frequently lack power to reach statistical significance. A recent meta‐analysis of 13 randomised clinical trials (mainly from the SURPASS and SURMOUNT programmes) evaluated the association between tirzepatide and cancer risk in individuals with and without T2D.^52^ Over 26–72 weeks in the full cohort, tirzepatide was associated with a non‐significant lower risk of cancer compared to placebo, insulin, or GLP‐1 receptor monoagonists (risk ratio: 0.78, 95% CI: 0.53–1.16), with similar findings in a subgroup analysis of individuals with T2D (risk ratio: 0.70, 95% CI: 0.44–1.12). Similarly, another meta‐analysis of four randomised clinical trials in people with T2D reported that over 40–72 weeks,^53^ tirzepatide was not associated with an increased risk of any type of cancer as compared to either insulin or placebo (risk ratio: 0.79, 95% CI: 0.24–2.56); however, when compared to placebo alone, a significantly lower risk of cancer was observed (0.35, 95% CI: 0.13–0.95).

The trend for a lower rate of cancer with tirzepatide versus DPP‐4i suggested by our study and other observational studies (albeit most lacking statistical power) builds on a growing number of preclinical in vivo studies that have shown an anti‐cancer effect of tirzepatide against a number of individual malignancies, including breast, endometrial and hepatocellular.^22^, ^25^ For example, using a transgenic mouse model of oestrogen, progesterone and PTEN (phosphatase and tensin homologue) deleted on chromosome 10 positive endometrial cancer, Kong et al. demonstrated that tirzepatide reduced endometrial tumour growth by ~60% compared to control, when animals were maintained on either a high or low‐fat diet to generate an obese or lean phenotype, respectively. Tirzepatide was associated with reduced body weight, with inhibition of tumour cell proliferation and increased apoptosis in both the lean and obese mice. Additionally, tirzepatide displayed distinct effects on metabolic and inflammatory pathways under both conditions (lean and obesity).^23^ Whilst tirzepatide, in our study, was not associated with a significantly lower rate of uterine (49% lower), liver (29% higher) or breast (4% lower) cancers, this may be related to a lack of statistical power. Notably, Kamrul‐Hasan et al. also reported a non‐significant reduction in breast cancer risk with tirzepatide compared to placebo in a meta‐analysis of RCTs.^52^ Another potential reason for the lack of rate reduction of individual OAC with tirzepatide use is the short duration of follow‐up (approximately 1 year), which may be too soon to detect any protective effects. Typically, most cancers take years or even decades to develop to a stage where they become symptomatic and/or clinically detectable through screening programmes. The lower rate of all OAC observed in the present study (although not significant) with tirzepatide after just ~1 year of follow‐up may represent an ability to suppress growing tumours rather than inhibit the formation of new lesions; future epidemiology and mechanistic studies will need to address whether the protective effects persist and at what stage(s) of the carcinogenic pathway tirzepatide acts in different populations.

#### 4.2.3 Bariatric surgery

Our study observed a statistically significant lower rate of OAC with BS versus DPP‐4i, which is consistent with several previous studies.^15^, ^54^, ^55^ Additionally, we observed a 41% lower rate of uterine cancer, aligning with prior research.^15^, ^55^ We also observed a 54% lower rate of liver cancer, which is broadly consistent with previous studies.^56^, ^57^, ^58^

We further observed an increase in gastric cardia and oesophageal cancer—although the actual numbers were very small (<10 events in both groups for each outcome), warranting cautious interpretation. In a large nationwide cohort study from France, which examined over 900,000 individuals (of which ≈300,000 have undergone BS), gastric cardia cancer appeared to be one of the frequent anatomical sites of gastric cancer following BS, with a trend towards increased incidence at this location.^59^ However, the study also reported a reduction in overall gastric cancer and a trend towards lower oesophageal cancer risk after BS, suggesting that the net effect of BS is probably protective with respect to upper gastrointestinal cancers.^59^

One possible explanation for the observed increase in gastric cardia cancer is surveillance bias. Gastrointestinal symptoms are common after BS, and patients may undergo more frequent upper gastrointestinal endoscopy than people without surgery.^60^ This may lead to more incidental detection of cancers in accessible sites such as the gastric pouch and cardia, particularly in symptomatic individuals.

Beyond surveillance bias, procedure‐specific mechanisms may also contribute to increased cancer risk at these sites after BS. Although assessing cancer risk by procedure type was beyond the scope of this study, the two most common procedures—Roux‐en‐Y gastric bypass (RYGB) and sleeve gastrectomy (SG)—alter gastrointestinal anatomy in different ways. SG is associated with increased gastroesophageal reflux, likely due to elevated intragastric pressure and disruption of the angle of His, exposing the cardia and oesophagus to acid and bile.^61^ This chronic exposure may promote inflammation, metaplasia (e.g., Barrett's oesophagus) and eventually cancer.

In contrast, RYGB typically reduces acid reflux by diverting food away from the acid‐producing stomach and duodenum. However, it creates a bypassed gastric remnant that remains anatomically intact but excluded from the digestive stream. This remnant can be exposed to bile and pancreatic secretions, which may promote chronic inflammation and carcinogenesis over time. Indeed, most gastric cancers reported after RYGB appear to occur in the gastric remnant—the excluded part of the stomach.^59^, ^62^ However, the small gastric pouch may still be exposed to bile reflux from the Roux limb in some patients. Although less common, this exposure could contribute to mucosal injury at the gastric cardia and may play a role in cancer development in this region.

### 4.3 WL and cancer risk

Although we did not measure change in body weight associated with each weight‐lowering intervention, overall, our findings support our hypothesis that WL may lower the rate of OAC. This may seem in contrast to a study of 157,474 health care professionals in which WL of >10% was associated with significantly higher risk of certain cancers such as upper gastrointestinal tract, colorectal, and lung cancers.^63^ It is difficult to compare these findings to our results for several reasons. Firstly, WL in our study was more likely to represent intentional weight reduction following intervention, whereas the study of health care professionals is likely to include individuals with unintentional WL, which may be due to underlying disease. Therefore, there was a risk of confounding or even reverse causality, from conditions such as cancer, causing WL prior to detection. Reverse causality is less likely to influence our results as our exposure was weight‐lowering interventions, rather than weight loss per se. Moreover, the follow‐up period in the earlier study (1988–2006) precedes the use of novel pharmacotherapies and, as discussed, these newer interventions may have weight‐independent cancer‐preventive effects.

### 4.4 Strengths and limitations

The strengths of this study include the utilisation of a large real‐world database, with a large number of individuals, allowing us to examine associations with new exposures such as tirzepatide. This study also utilised objectively measured BMI and HbA1c among numerous other confounders for propensity score matching.

The limitations of this study include an unknown degree of missing data, unknown completeness of cancer diagnosis recording, and the variables used for propensity score matching. This might have led to residual confounding. Moreover, the follow‐up, especially for the tirzepatide and semaglutide groups, was short (1–2 years), with the duration of treatment and dosages used for these medications also unknown. TriNetX data does not capture death out‐of‐hospital, and death cannot be used for right censoring within the platform. Although we matched our cohorts with codes for potential health hazards related to socioeconomic and psychosocial circumstances, more granular socio‐economic factors were not available for inclusion as potential confounders. Diabetes duration was also not available for matching. Another limitation was the difference in cohort sizes; semaglutide has a much larger cohort, which may have contributed to differences in statistical power regarding cancer outcomes. Furthermore, there may be bias in the recording and definition of cancer used in our study. We used clinical codes for cancer diagnosis, which were extracted from medical records and harmonised onto the TriNetX platform; however, the diagnostic criteria for cancers applied within each contributing HCOs are unknown, meaning there is a risk of outcome misclassification. Despite including a diverse ethnic sample, the data are exclusively from the USA, limiting the study's applicability to other populations. Lastly, we were unable to adjust our threshold for significance to account for multiple testing due to limitations of the TriNetX platform, meaning our findings are at risk of type I error and that ‘statistically significant’ findings should therefore be interpreted with caution.

## 5 CONCLUSION

Our findings demonstrate that among individuals with T2D and overweight/obesity, semaglutide and BS were both associated with a significantly lower rate of composite OAC as compared to DPP‐4i, with a non‐significant lower rate also seen with tirzepatide. These results suggest that WL interventions in people with T2D and overweight/obesity may have a role in prevention strategies for OAC compared to DPP‐4i. Further long‐term studies are needed to explore the potential cancer‐preventive effects of GLP‐1 RA‐based therapies and BS—both in OAC and non‐OAC settings—along with the underlying mechanisms for people with and without T2D. Additionally, emerging pharmacotherapies for overweight/obesity and T2D, such as dual and triple agonists targeting multiple entero‐pancreatic hormone pathways represent an area for future research in cancer prevention.

## CONFLICT OF INTEREST STATEMENT

D.P. has received honoraria for lectures from Novo Nordisk, Eli Lilly, Boehringer Ingelheim and Johnson & Johnson; consultancy fees from Regeneron; and research grants from Novo Nordisk, the Novo Nordisk UK Research Foundation, the Academy of Medical Sciences/Diabetes UK, and the National Institute for Health and Care Research (NIHR). MJD has acted as a consultant/advisor and speaker for Eli Lilly, Novo Nordisk and Sanofi, has attended advisory boards for AbbVie, Amgen, AstraZeneca, Biomea Fusion, Carmot/Roche, Daewoong Pharmaceutical, Sanofi, Zealand Pharma, Regeneron, GSK and EktaH, and as a speaker for AstraZeneca, Boehringer Ingelheim and Zuellig Pharma. She has received grants from AstraZeneca, Boehringer Ingelheim and Novo Nordisk. TJW has received consulting fees from Regeneron and Baxter Int. FZ has received consulting fees from Servier, Menarini and Daiichi Sankyo. TY has received investigated initiated funding from Astra Zeneca, has acted as a consultant for Regeneron, and has received contacted research funding from the Reinsurance Group of America.

## PEER REVIEW

The peer review history for this article is available at https://www.webofscience.com/api/gateway/wos/peer-review/10.1111/dom.70090.

## Supporting information


**Data S1.** Supporting information.

## Data Availability

The data that support the findings of this study are available from TriNetX. Restrictions apply to the availability of these data, which were used under license for this study. Data are available from the authors with the permission of TriNetX.

## References

[1] Keys A , Fidanza F , Karvonen MJ , Kimura N , Taylo HL . Indices of relative weight and obesity. J Chronic Dis. 1972;19(25):329‐343. 10.1016/0021-9681(72)90027-6 4650929

[2] WorldHealthOrganization . Obesity. World Health Organization. Accessed November 19, 2024. https://www.who.int/news-room/fact-sheets/detail/obesity-and-overweight

[3] GBD 2015 Obesity Collaborators , Afshin A , Forouzanfar MH , et al. Health effects of overweight and obesity in 195 countries over 25 years. N Engl J Med. 2017;377(1):13‐27. 28604169 10.1056/NEJMoa1614362 PMC5477817

[4] Jensen MD , Ryan DH , Apovian CM , et al. 2013 AHA/ACC/TOS guideline for the management of overweight and obesity in adults: a report of the American College of Cardiology/American Heart Association Task Force on Practice Guidelines and The Obesity Society. J Am Coll Cardiol. 2014;63(25 Pt B):2985‐3023. doi:10.1016/j.jacc.2013.11.004 24239920

[5] Di Angelantonio E , Bhupathiraju SN , Wormser D , et al. Body‐mass index and all‐cause mortality: individual‐participant‐data meta‐analysis of 239 prospective studies in four continents. The Lancet. 2016;388(10046):776‐786. doi:10.1016/s0140-6736(16)30175-1 PMC4995441 27423262

[6] Kim D‐S , Scherer PE . Obesity, Diabetes, and increased cancer progression. Diabetes Metab J. 2021;45(6):799‐812. doi:10.4093/dmj.2021.0077 34847640 PMC8640143

[7] Scully T , Ettela A , Leroith D , Gallagher EJ . Obesity, type 2 Diabetes, and cancer risk. Front Oncol. 2021;10:615375. doi:10.3389/fonc.2020.615375 33604295 PMC7884814

[8] Pearson‐Stuttard J , Zhou B , Kontis V , Bentham J , Gunter MJ , M E . Worldwide burden of cancer attributable to diabetes and high body‐mass index: a comparative risk assessment. Lancet Diabetes Endocrinol. 2018;6:e6‐e15. 10.1016/S2213-8587(18)30150-5 PMC5982644 29803268

[9] Lauby‐Secretan B , Scoccianti C , Loomis D , Grosse Y , Bianchini F , Straif K . Body fatness and cancer—viewpoint of the IARC working group. N Engl J Med. 2016;375(8):794‐798. doi:10.1056/nejmsr1606602 27557308 PMC6754861

[10] Cohen DH , Leroith D . Obesity, type 2 diabetes, and cancer: the insulin and IGF connection. Endocr Relat Cancer. 2012;19(5):F27‐F45. doi:10.1530/erc-11-0374 22593429

[11] Ling S , Zaccardi F , Issa E , Davies MJ , Khunti K , Brown K . Inequalities in cancer mortality trends in people with type 2 diabetes: 20 year population‐based study in England. Diabetologia. 2023;66(4):657‐673. doi:10.1007/s00125-022-05854-8 36690836 PMC9947024

[12] Pearson‐Stuttard J , Bennett J , Cheng YJ , et al. Trends in predominant causes of death in individuals with and without diabetes in England from 2001 to 2018: an epidemiological analysis of linked primary care records. Lancet Diabetes Endocrinol. 2021;9(3):165‐173. doi:10.1016/s2213-8587(20)30431-9 33549162 PMC7886654

[13] Tomic D , Chen L , Moran LL , Magliano DJ , Shaw JE . Causes of death among Australians with type 1 or type 2 diabetes, 2002–2019. Diabet Med. 2024;41(3):e15206. doi:10.1111/dme.15206 37597240

[14] Chittajallu V , Mansoor E , Perez J , et al. Association of bariatric surgery with risk of incident obesity‐associated malignancies: a multi‐center population‐based study. Obes Surg. 2023;33(12):4065‐4069. doi:10.1007/s11695-023-06926-3 37971573

[15] Aminian A , Wilson R , Al‐Kurd A , et al. Association of bariatric surgery with cancer risk and mortality in adults with obesity. Jama. 2022;327(24):2423‐2433. doi:10.1001/jama.2022.9009 35657620 PMC9166218

[16] Maciejewski ML , Arterburn DE , Van Scoyoc L , et al. Bariatric surgery and long‐term durability of weight loss. JAMA Surg. 2016;151(11):1046‐1055. doi:10.1001/jamasurg.2016.2317 27579793 PMC5112115

[17] Ram Sohan P , Mahakalkar C , Kshirsagar S , et al. Long‐term effectiveness and outcomes of bariatric surgery: a comprehensive review of current evidence and emerging trends. Cureus. 2024;16:e66500. doi:10.7759/cureus.66500 39247032 PMC11381104

[18] Colin IM , Gérard A‐C . Once‐weekly 2.4 mg semaglutide for weight management in obesity: a game changer? Endocrinology. 2022;18(1):35. doi:10.17925/ee.2022.18.1.35 PMC9354513 35949360

[19] Davies M , Faerch L , Jeppesen OK , et al. Semaglutide 2.4 mg once a week in adults with overweight or obesity, and type 2 diabetes (STEP 2): a randomised, double‐blind, double‐dummy, placebo‐controlled, phase 3 trial. Lancet. 2021;397(10278):971‐984. doi:10.1016/S0140-6736(21)00213-0 33667417

[20] Garvey WT , Frias JP , Jastreboff AM , et al. Tirzepatide once weekly for the treatment of obesity in people with type 2 diabetes (SURMOUNT‐2): a double‐blind, randomised, multicentre, placebo‐controlled, phase 3 trial. Lancet. 2023;402(10402):613‐626. doi:10.1016/s0140-6736(23)01200-x 37385275

[21] Liu Y , Chen S , Zhen R . Effect of Semaglutide on high‐fat‐diet‐induced liver cancer in obese mice. J Proteome Res. 2024;23(2):704‐717. doi:10.1021/acs.jproteome.3c00498 38227547 PMC10846501

[22] Glenny EM , Ho AN , Kiesel VA , et al. Tirzepatide attenuates mammary tumor progression in diet‐induced obese mice. bioRxiv. 2024;2024.01.20.576484. doi:10.1101/2024.01.20.576484

[23] Kong W , Deng B , Shen X , et al. Tirzepatide as an innovative treatment strategy in a pre‐clinical model of obesity‐driven endometrial cancer. Gynecol Oncol. 2024;191:116‐123. doi:10.1016/j.ygyno.2024.10.004 39388742 PMC12419173

[24] Liu Y , Ban J , Yang L , et al. Effect of High‐Fat‐Diet and Semaglutide on Bladder Cancer in Mice. Springer Science and Business Media LLC; 2024.

[25] Jeong BK , Choi WI , Choi W , et al. A male mouse model for metabolic dysfunction‐associated steatotic liver disease and hepatocellular carcinoma. Nat Commun. 2024;15(1):6506. doi:10.1038/s41467-024-50660-y 39090079 PMC11294468

[26] Li Q , Tuo X , Li B , Deng Z , Qiu Y , Xie H . Semaglutide attenuates excessive exercise‐induced myocardial injury through inhibiting oxidative stress and inflammation in rats. Life Sci. 2020;250:117531. doi:10.1016/j.lfs.2020.117531 32151691

[27] Popoviciu M‐S , Păduraru L , Yahya G , Metwally K , Cavalu S . Emerging role of GLP‐1 agonists in obesity: a comprehensive review of randomised controlled trials. Int J Mol Sci. 2023;24(13):10449. doi:10.3390/ijms241310449 37445623 PMC10341852

[28] Neumiller JJ . Incretin‐based therapies. Med Clin North Am. 2015;99(1):107‐129. doi:10.1016/j.mcna.2014.08.013 25456646

[29] Diabetes UK . DPP‐4 inhibitors (gliptins). Diabetes UK. https://www.diabetes.org.uk/about-diabetes/looking-after-diabetes/treatments/tablets-and-medication/dpp-4-inhibitors-gliptins

[30] Zhao M , Chen J , Yuan Y , et al. Dipeptidyl peptidase‐4 inhibitors and cancer risk in patients with type 2 diabetes: a meta‐analysis of randomized clinical trials. Sci Rep. 2017;7(1):8273. doi:10.1038/s41598-017-07921-2 28811622 PMC5557948

[31] Palchuk MB , London JW , Perez‐Rey D , et al. A global federated real‐world data and analytics platform for research. JAMIA Open. Jul 2023;6(2):ooad035. doi:10.1093/jamiaopen/ooad035 37193038 PMC10182857

[32] Gasoyan H , Butsch WS , Schulte R , et al. Changes in weight and glycemic control following obesity treatment with semaglutide or tirzepatide by discontinuation status. Obesity. 2025;33(9):1657‐1667. doi:10.1002/oby.24331 40491239 PMC12381620

[33] Rodriguez PJ , Zhang V , Gratzl S , et al. Discontinuation and reinitiation of dual‐labeled GLP‐1 receptor agonists among US adults with overweight or obesity. JAMA Netw Open. 2025;8(1):e2457349. doi:10.1001/jamanetworkopen.2024.57349 39888616 PMC11786232

[34] Rubino D , Abrahamsson N , Davies M , et al. Effect of continued weekly subcutaneous Semaglutide vs placebo on weight loss maintenance in adults with overweight or obesity. Jama. 2021;325(14):1414. doi:10.1001/jama.2021.3224 33755728 PMC7988425

[35] Hansen HH , Pors S , Andersen MW , et al. Semaglutide reduces tumor burden in the GAN diet‐induced obese and biopsy‐confirmed mouse model of NASH‐HCC with advanced fibrosis. Sci Rep. 2023;13(1):23056. doi:10.1038/s41598-023-50328-5 38155202 PMC10754821

[36] Miousse IR . GLP‐1 receptor agonists in the context of cancer: the road ahead. Am J Physiol Cell Physiol. 2025;328(6):C1822‐C1828. doi:10.1152/ajpcell.00245.2025 40285503 PMC12109175

[37] Ryan DH , Lingvay I , Deanfield J , et al. Long‐term weight loss effects of semaglutide in obesity without diabetes in the SELECT trial. Nat Med. 2024;30(7):2049‐2057. doi:10.1038/s41591-024-02996-7 38740993 PMC11271387

[38] Sjöholm K , Carlsson LMS , Svensson P‐A , et al. Association of bariatric surgery with cancer incidence in patients with obesity and diabetes: long‐term results from the Swedish obese subjects study. Diabetes Care. 2022;45(2):444‐450. doi:10.2337/dc21-1335 34799430 PMC8914410

[39] Stroud AM , Coleman MF . Bariatric surgery in the prevention of obesity‐associated cancers: mechanistic implications. Surg Obes Relat Dis. 2023;19(7):772‐780. 37120355 10.1016/j.soard.2023.02.016

[40] Bezin J , Gouverneur A , Pénichon M , et al. GLP‐1 receptor agonists and the risk of thyroid cancer. Diabetes Care. 2023;46(2):384‐390. doi:10.2337/dc22-1148 36356111

[41] Silverii GA , Monami M , Gallo M , et al. Glucagon‐like peptide‐1 receptor agonists and risk of thyroid cancer: a systematic review and meta‐analysis of randomized controlled trials. Diabetes Obes Metab. 2024;26(3):891‐900. doi:10.1111/dom.15382 38018310

[42] Elashoff M , Matveyenko AV , Gier B , Elashoff R , Butler PC . Pancreatitis, pancreatic, and thyroid cancer with glucagon‐like Peptide‐1–based therapies. Gastroenterology. 2011;141(1):150‐156. doi:10.1053/j.gastro.2011.02.018 21334333 PMC4404515

[43] Wang L , Wang Q , Li L , Kaelber DC , Xu R . Glucagon‐like peptide‐1 receptor agonists and pancreatic cancer risk: target trial emulation using real‐world data. J Natl Cancer Inst. 2024;117:476‐485. doi:10.1093/jnci/djae260 PMC11884861 39418202

[44] Baxter SM , Lund LC , Andersen JH , et al. Glucagon‐like peptide 1 receptor agonists and risk of thyroid cancer: an international multisite cohort study. Thyroid. 2025;35(1):69‐78. doi:10.1089/thy.2024.0387 39772758

[45] Nagendra L , Bg H , Sharma M , Dutta D . Semaglutide and cancer: a systematic review and meta‐analysis. Diabetes Metab Syndr. 2023;17(9):102834. doi:10.1016/j.dsx.2023.102834 37531876

[46] Arunkumar K , Yousaf H , William H , Shyam T , Shailendra S . Glucagon‐like peptide 1–based therapies and risk of pancreatic cancer in patients with diabetes and obesity. Pancreas. 2022;50(10):1398‐1403. doi:10.1097/MPA.0000000000002197 37099785

[47] Wang L , Xu R , Kaelber DC , Berger NA . Glucagon‐like peptide 1 receptor agonists and 13 obesity‐associated cancers in patients with type 2 diabetes. JAMA Netw Open. 2024;7(7):e2421305. doi:10.1001/jamanetworkopen.2024.21305 38967919 PMC11227080

[48] Krause D , Chandra V , Dey DK . Effect of GLP‐1 receptor agonist therapy on endometrial cancer. Gynecol Oncol. 2024;190:S431‐S459.

[49] Stanisavljevic I , Pavlovic S , Simovic Markovic B , et al. Semaglutide decelerates the growth and progression of breast cancer by enhancing the acquired antitumor immunity. Biomed Pharmacother. 2024;181:117668. doi:10.1016/j.biopha.2024.117668 39536536

[50] Frías JP , Davies MJ , Rosenstock J , et al. Tirzepatide versus Semaglutide once weekly in patients with type 2 diabetes. N Engl J Med. 2021;385(6):503‐515. doi:10.1056/nejmoa2107519 34170647

[51] Thomas MK , Nikooienejad A , Bray R , et al. Dual GIP and GLP‐1 receptor agonist tirzepatide improves beta‐cell function and insulin sensitivity in type 2 diabetes. J Clin Endocrinol Metabol. 2021;106(2):388‐396. doi:10.1210/clinem/dgaa863 PMC7823251 33236115

[52] Kamrul‐Hasan ABM , Alam MS , Dutta D , Sasikanth T , Aalpona FTZ , Nagendra L . Tirzepatide and cancer risk in individuals with and without diabetes: a systematic review and meta‐analysis. Endocrinol Metab (Seoul). 2025;40(1):112‐124. doi:10.3803/EnM.2024.2164 39814031 PMC11898313

[53] Popovic DS , Patoulias D , Popovic LS , Karakasis P , Papanas N , Mantzoros CS . Tirzepatide use and the risk of cancer among individuals with type 2 diabetes mellitus: a meta‐analysis of randomized controlled trials. Diabetes Res Clin Pract. 2024;213:111758. doi:10.1016/j.diabres.2024.111758 38925294

[54] Wiggins T , Antonowicz SS , Markar SR . Cancer risk following bariatric surgery—systematic review and meta‐analysis of national population‐based cohort studies. Obes Surg. 2019;29(3):1031‐1039. doi:10.1007/s11695-018-3501-8 30591985

[55] Schauer DP , Feigelson HS , Koebnick C , et al. Bariatric surgery and the risk of cancer in a large multisite cohort. Ann Surg. 2019;269(1):95‐101. doi:10.1097/sla.0000000000002525 28938270 PMC6201282

[56] Rustgi VK , Li Y , Gupta K , et al. Bariatric surgery reduces cancer risk in adults with nonalcoholic fatty liver disease and severe obesity. Gastroenterology. 2021;161(1):171‐184.e10. doi:10.1053/j.gastro.2021.03.021 33744305

[57] Yang B , Yang HP , Ward KK , Sahasrabuddhe VV , McGlynn KA . Bariatric surgery and liver cancer in a consortium of academic medical centers. Obes Surg. 2016;26(3):696‐700. doi:10.1007/s11695-016-2051-1 26757918 PMC4769957

[58] Ramai D , Singh J , Lester J , et al. Systematic review with meta‐analysis: bariatric surgery reduces the incidence of hepatocellular carcinoma. Aliment Pharmacol Ther. 2021;53(9):977‐984. doi:10.1111/apt.16335 33721336

[59] Lazzati A , Poghosyan T , Touati M , Collet D , Gronnier C . Risk of esophageal and gastric cancer after bariatric surgery. JAMA Surg. 2023;158(3):264‐271. doi:10.1001/jamasurg.2022.6998 36630108 PMC9857712

[60] Brown WA , Johari Halim Shah Y , Balalis G , et al. IFSO position statement on the role of esophago‐gastro‐duodenal endoscopy prior to and after bariatric and metabolic surgery procedures. Obes Surg. 2020;30(8):3135‐3153. doi:10.1007/s11695-020-04720-z 32472360

[61] DuPree CE , Blair K , Steele SR , Martin MJ . Laparoscopic sleeve gastrectomy in patients with preexisting gastroesophageal reflux disease: a national analysis. JAMA Surg. 2014;149(4):328‐334. doi:10.1001/jamasurg.2013.4323 24500799

[62] Youk KM , Kim J , Cho Y‐S , Park DJ . Gastric cancer after bariatric surgeries. Journal of Metabolic and Bariatric Surgery. 2022;11(2):20‐29. doi:10.17476/jmbs.2022.11.2.20 36926673 PMC10011677

[63] Wang Q‐L , Babic A , Rosenthal MH , et al. Cancer diagnoses after recent weight loss. Jama. 2024;331(318–328):4‐328. doi:10.1001/jama.2023.25869 PMC10807298 38261044

