# Supplementary material for: Weight loss interventions and obesity‐associated cancers in people with type 2 diabetes and overweight/obesity: A real‐world observational study
Source: Diabetes Obes Metab. 2025 Sep 3;27(12):6914–26. doi: 10.1111/dom.70090 (PMC12587238; doi:10.1111/dom.70090)
Supplement: Supplementary file 1 — Data S1. Supporting information. [file DOM-27-6914-s001.pdf]

Supplementary Appendix

WEIGHT LOSS INTERVENTIONS AND OBESITY-RELATED CANCER IN PEOPLE WITH TYPE 2 DIABETES AND OVERWEIGHT/OBESITY: A REAL-WORLD OBSERVATIONAL STUDY

Testimony Ipaye, Jonathan Goldney, Thomas J. Wilkinson, Francesco Zaccardi, Thomas Yates, Melanie J. Davies, Karen Brown, Dimitris Papamargaritis

Contents

Supplementary Table S1: Procedure codes used to identify people undergoing bariatric surgery 2

Supplementary Table S2: Clinical codes used to define outcomes 3

Supplementary Table S3: Clinical codes used for diagnoses used in propensity score matching 4

Supplementary Table S4: Baseline characteristics of patients identified in the semaglutide, tirzepatide and BS cohort before propensity score matching 5

Supplementary Table S5: Follow-up time 7

Supplementary Figure S1: Sensitivity analysis excluding individuals that developed OAC within six months of follow-up 8

The RECORD statement 9

Supplementary Table S1: Procedure codes used to identify people undergoing bariatric surgery

Coding system	Reference	Code name
**CPT**	43644	Laparoscopy, surgical, gastric restrictive procedure; with gastric bypass and Roux-en-Y gastroenterostomy (roux limb 150 cm or less)
	43645	Laparoscopy, surgical, gastric restrictive procedure; with gastric bypass and small intestine reconstruction to limit absorption
	43770	Laparoscopy, surgical, gastric restrictive procedure; placement of adjustable gastric restrictive device (eg, gastric band and subcutaneous port components)
	43775	Laparoscopy, surgical, gastric restrictive procedure; longitudinal gastrectomy (ie, sleeve gastrectomy)
	43842	Gastric restrictive procedure, without gastric bypass, for morbid obesity; vertical-banded gastroplasty
	43843	Gastric restrictive procedure, without gastric bypass, for morbid obesity; other than vertical-banded gastroplasty
	43845	Gastric restrictive procedure with partial gastrectomy, pylorus-preserving duodenoileostomy and ileoileostomy (50 to 100 cm common channel) to limit absorption (biliopancreatic diversion with duodenal switch)
	43846	Gastric restrictive procedure, with gastric bypass for morbid obesity; with short limb (150 cm or less) Roux-en-Y gastroenterostomy
	43847	Gastric restrictive procedure, with gastric bypass for morbid obesity; with small intestine reconstruction to limit absorption
	1007403	Gastric restrictive procedure, without gastric bypass, for morbid obesity
	1014146	Gastric restrictive procedure, with gastric bypass for morbid obesity
**ICD-10-PCS**	0D1607A	Bypass Stomach to Jejunum with Autologous Tissue Substitute, Open Approach
	0D160JA	Bypass Stomach to Jejunum with Synthetic Substitute, Open Approach
	0D160KA	Bypass Stomach to Jejunum with Nonautologous Tissue Substitute, Open Approach
	0D160ZA	Bypass Stomach to Jejunum, Open Approach
	0D1687A	Bypass Stomach to Jejunum with Autologous Tissue Substitute, Via Natural or Artificial Opening Endoscopic
	0D168JA	Bypass Stomach to Jejunum with Synthetic Substitute, Via Natural or Artificial Opening Endoscopic
	0D168KA	Bypass Stomach to Jejunum with Nonautologous Tissue Substitute, Via Natural or Artificial Opening Endoscopic
	0D168ZA	Bypass Stomach to Jejunum, Via Natural or Artificial Opening Endoscopic
	0DV64CZ	Restriction of Stomach with Extraluminal Device, Percutaneous Endoscopic Approach
**SNOMED CT**	30803004	Printen and Mason operation, high gastric bypass
	173747005	Roux-en-Y gastrojejunostomy
	426738005	Duodenal switch
	427074001	Laparoscopic sleeve gastrectomy

	427980007	Sleeve gastrectomy with duodenal switch

CPT: Current Procedural Terminology; **ICD-10-PCD:** International Classification of Diseases Procedure Coding System (10th edition); **SNOMED CT:** Systematized Nomenclature of Medicine Clinical Terms

Supplementary Table S2: Clinical codes used to define outcomes

Outcome	ICD-10-CM code	Code name
**Breast cancer**	C59	Malignant neoplasm of breast
**Colorectal cancer**	C18	Malignant neoplasm of colon
	C19	Malignant neoplasm of rectosigmoid junction
	C20	Malignant neoplasm of rectum
**Gallbladder cancer**	C23	Malignant neoplasm of gallbladder
**Liver cancer**	C22	Malignant neoplasm of liver and intrahepatic bile ducts
**Multiple myeloma**	C90	Multiple myeloma and malignant plasma cell neoplasms
**Oesophageal cancer**	C15	Malignant neoplasm of oesophagus
**Ovarian cancer**	C56	Malignant neoplasm of ovary
**Pancreatic cancer**	C25	Malignant neoplasm of pancreas
**Renal cancer**	C64	Malignant neoplasm of kidney, except renal pelvis
**Gastric cardia cancer**	C16.0	Malignant neoplasm of cardia
**Thyroid cancer**	C73	Malignant neoplasm of thyroid gland
**Uterine cancer**	C54	Malignant neoplasm of corpus uteri

ICD-10-CM: International Classification of Diseases Clinical Modification (10th revision)

Supplementary Table S3: Clinical codes used for diagnoses used in propensity score matching

ICD-10-CM code	Code name
E11.2	Type 2 diabetes mellitus with kidney complications
E11.3	Type 2 diabetes mellitus with ophthalmic complications
E11.4	Type 2 diabetes mellitus with neurological complications
F10	Alcohol related disorders
F17	Nicotine dependence
I10-I1A	Hypertensive diseases
I20-I25	Ischemic heart diseases
I60-I69	Cerebrovascular diseases
I70	Atherosclerosis
I73.9	Peripheral vascular disease, unspecified
Z87.891	Personal history of nicotine dependence
Z55-Z65*	Persons with potential health hazards related to socioeconomic and psychosocial circumstances*

ICD-10-CM: International Classification of Diseases Clinical Modification (10th revision)

*Z55-Z65 includes: Z55 Problems related to education and literacy; Z56 Problems related to employment and unemployment; Z57 Occupational exposure to risk-factors; Z58 Problems related to physical environment; Z59 Problems related to housing and economic circumstances; Z60 Problems related to social environment; Z61 Problems related to negative life events in childhood; Z62 Other problems related to upbringing; Z63 Other problems related to primary support group, including family circumstances; Z64 Problems related to certain psychosocial circumstances; Z65 Problems related to other psychosocial circumstances. A more granular breakdown of these codes can be found at https://icd.who.int/browse10/2019/en/

Supplementary Table S4: Baseline characteristics of patients identified in the semaglutide, tirzepatide and BS cohort before propensity score matching

		Semaglutide vs DPP-4i		Tirzepatide vs DPP-4i		Bariatric surgery vs DPP-4i	
		**Exposure**	**Comparator (DPP-4i)**	**Exposure**	**Comparator (DPP-4i)**	**Exposure**	**Comparator (DPP-4i)**
**People**		170,273	188,567	45,047	188,567	15,911	188,567
**Current Age**		58.8 (12.7)	67.7 (12.3)	56.5 (12.7)	67.7 (12.3)	53.6 (12.8)	67.7 (12.3)
**Age at Index**		56.6 (12.8)	61.0 (12.3)	55.3 (12.7)	61.0 (12.3)	47.5 (11.9)	61.0 (12.3)
**Sex**	**Female**	89339 (52.4)	86184 (45.7)	23704 (52.6)	86184 (45.7)	11525 (72.8)	86184 (45.7)
	**Male**	75130 (44.1)	97259 (51.5)	20114 (44.6)	97259 (51.5)	3743 (23.6)	97259 (51.5)
	**Unknown**	5804 (3.40)	5091 (2.7)	1229 (2.72)	5091 (2.7)	549 (3.47)	5091 (2.744)
**Race**	**American Indian or Alaska Native**	1021 (0.6)	931 (0.49)	199 (0.44)	931 (0.49)	69 (0.43)	931 (0.49)
	**Asian**	6541 (3.84)	10388 (5.51)	1335 (2.96)	10388 (5.51)	144 (0.91)	10388 (5.51)
	**Black or African American**	29784 (17.4)	37415 (19.8)	6912 (15.3)	37415 (19.8)	3467 (21.9)	37415 (19.8)
	**Native Hawaiian or Other Pacific Islander**	1643 (0.96)	2570 (1.36)	276 (0.61)	2570 (1.36)	56 (0.35)	2570 (1.36)
	**White**	111161 (65.2)	113747 (60.3)	31205 (69.2)	113747 (60.3)	9661 (61.0)	113747 (60.3)
	**Other Race**	6292 (3.69)	7452 (3.95)	1696 (3.76)	7452 (3.95)	820 (5.18)	7452 (3.95)
	**Unknown Race**	13831 (8.12)	16031 (8.50)	3424 (7.60)	16031 (8.50)	1600 (10.1)	16031 (8.50)
**Ethnicity**	**Hispanic or Latino**	15546 (9.13)	17794 (9.43)	4003 (8.88)	17794 (9.43)	2599 (16.4)	17794 (9.43)
	**Not Hispanic or Latino**	114125 (67.0)	139214 (73.8)	29510 (65.5)	139214 (73.8)	10815 (68.3)	139214 (73.8)
	**Unknown Ethnicity**	40602 (23.8)	31526 (16.7)	11534 (25.6)	31526 (16.7)	2403 (15.1)	31526 (16.7)
**BMI kg/m^2**		37.3 (7.82)	33.5 (7.00)	38.3 (7.84)	33.5 (7.00)	44.9 (8.21)	33.5 (7.00)
**BMI categories**	**25-30 kg/m^2**	34752 (20.4)	75303 (39.9)	7158 (15.8)	75303 (39.9)	448 (2.83)	75303 (39.9)
	**30-35 kg/m2**	61034 (35.8)	76024 (40.3)	15095 (33.5)	76024 (40.3)	1145 (7.23)	76024 (40.3)
	**35-40 kg/m2**	57433 (33.7)	48585 (25.7)	15737 (34.9)	48585 (25.7)	4710 (29.7)	48585 (25.7)
	**40-45 kg/m2**	38417 (22.5)	25555 (13.5)	11183 (24.8)	25555 (13.5)	6741 (42.6)	25555 (13.5)
	**45-50 kg/m2**	21632 (12.7)	12157 (6.44)	6197 (13.7)	12157 (6.44)	5775 (36.5)	12157 (6.44)
	**50-55 kg/m2**	10948 (6.43)	5492 (2.91)	3276 (7.27)	5492 (2.91)	3951 (24.9)	5492 (2.91)
	**>55 kg/m2**	7230 (4.24)	3694 (1.95)	2197 (4.87)	3694 (1.95)	3171 (20.0)	3694 (1.95)
**HbA1c** %		7.80 (1.93)	8.24 (1.90)	7.52 (1.90)	8.24 (1.90)	6.60 (1.28)	8.24 (1.90)
**HbA1c categories**	**<6.5 %**	63142 (37.0)	41656 (22.0)	19932 (44.2)	41656 (22.0)	10033 (63.4)	41656 (22.0)
	**6.5-7.5 %**	71250 (41.8)	78598 (41.6)	18928 (42.0)	78598 (41.6)	5554 (35.1)	78598 (41.6)
	**7.5-8.5 %**	43359 (25.4)	65866 (34.9)	9141 (20.2)	65866 (34.9)	2409 (15.2)	65866 (34.9)
	**8.5-9.5 %**	26522 (15.5)	39927 (21.1)	5209 (11.5)	39927 (21.1)	1165 (7.36)	39927 (21.1)
	**9.5-10.5 %**	17013 (9.99)	23549 (12.4)	3469 (7.70)	23549 (12.4)	625 (3.95)	23549 (12.4)
	**10.5-11.5 %**	11907 (6.99)	14967 (7.93)	2472 (5.48)	14967 (7.93)	305 (1.92)	14967 (7.93)
	**>11.5 %**	14664 (8.61)	18411 (9.76)	3314 (7.35)	18411 (9.76)	315 (1.99)	18411 (9.76)
**Past medical history**	**Hypertensive diseases**	122053 (71.6)	146504 (77.7)	30660 (68.0)	146504 (77.7)	11926 (75.4)	146504 (77.7)
	**Ischemic heart diseases**	26883 (15.7)	40298 (21.3)	5847 (12.9)	40298 (21.3)	1800 (11.3)	40298 (21.3)

	**Type 2 diabetes mellitus with kidney complications**	20636 (12.1)	30627 (16.2)	4363 (9.68)	30627 (16.2)	1040 (6.57)	30627 (16.2)
	**Type 2 diabetes mellitus with neurological complications**	18354 (10.7)	22957 (12.1)	4142 (9.19)	22957 (12.1)	1121 (7.08)	22957 (12.1)
	**Nicotine dependence**	15669 (9.20)	18757 (9.94)	3530 (7.83)	18757 (9.94)	1447 (9.14)	18757 (9.94)
	**Cerebrovascular diseases**	8786 (5.16)	17628 (9.35)	1883 (4.18)	17628 (9.35)	443 (2.80)	17628 (9.35)
	**Type 2 diabetes mellitus with ophthalmic complications**	6570 (3.85)	8848 (4.69)	1437 (3.19)	8848 (4.69)	320 (2.02)	8848 (4.69)
	**Atherosclerosis**	5261 (3.09)	8178 (4.33)	1224 (2.71)	8178 (4.33)	212 (1.34)	8178 (4.33)
	**Personal history of nicotine dependence**	19460 (11.4)	20787 (11.0)	4690 (10.4)	20787 (11.0)	3787 (23.9)	20787 (11.0)
	**Peripheral vascular disease, unspecified**	4787 (2.81)	8056 (4.27)	1029 (2.28)	8056 (4.27)	232 (1.46)	8056 (4.27)
	**Alcohol related disorders**	3124 (1.83)	3773 (2.00)	833 (1.84)	3773 (2.00)	331 (2.09)	3773 (2.00)
**Glucose-lowering therapies**	**Metformin**	87661 (51.4)	117831 (62.4)	19912 (44.2)	117831 (62.4)	5226 (33.0)	117831 (62.4)
	**Insulin**	42133 (24.7)	71604 (37.9)	9166 (20.3)	71604 (37.9)	8208 (51.8)	71604 (37.9)
	**SGLT2i**	29700 (17.4)	20679 (10.9)	6917 (15.3)	20679 (10.9)	423 (2.67)	20679 (10.9)
	**Sulfonylureas**	23012 (13.5)	57858 (30.6)	4179 (9.27)	57858 (30.6)	1057 (6.68)	57858 (30.6)
**Persons with potential health hazards related to socioeconomic and psychosocial circumstances**		4733 (2.78)	4205 (2.23)	1339 (2.97)	4205 (2.23)	559 (3.53)	4205 (2.23)

Supplementary Table S5: Follow-up time

Analysis	Cohort	Mean follow-up time (days)	Standard deviation	Median follow-up time (days)	Interquartile range
Semaglutide vs. DPP-4i	Semaglutide	911	557	846	806
	DPP-4i	864	666	766	1011
Tirzepatide vs. DPP-4i	Tirzepatide	435	279	421	477
	DPP-4i	439	389	345	563
Bariatric surgery vs. DPP-4i	Bariatric surgery	1746	1395	1421	1826
	DPP-4i	1823	1358	1597	1951

DPP-4i: Dipeptidyl peptidase-4 inhibitor

Supplementary Figure S6: Sensitivity analysis excluding individuals that developed OAC within six months of follow-up

Exposure	Outcome	Exposure People	Exposure Events (%)	Comparator People	Comparator Events (%)	Hazard ratio with 95% CI
Semaglutide	Composite cancer	63671	1142 (1.79)	63671	1091 (1.71)	1.00 [0.92, 1.09]
	Breast cancer	63671	311 (0.49)	63671	316 (0.50)	0.95 [0.81, 1.11]
	Colorectal cancer	63671	175 (0.27)	63671	180 (0.28)	0.94 [0.76, 1.16]
	Gallbladder cancer	63671	<10 (0.02)	63671	<10 (0.02)	0.67 [0.19, 2.38]
	Liver cancer	63671	111 (0.17)	63671	110 (0.17)	0.97 [0.74, 1.26]
	Multiple myeloma	63671	75 (0.12)	63671	59 (0.09)	1.21 [0.86, 1.70]
	Oesophageal cancer	63671	28 (0.04)	63671	24 (0.04)	1.15 [0.66, 1.99]
	Ovarian cancer	63671	41 (0.06)	63671	33 (0.05)	1.20 [0.76, 1.91]
	Pancreatic cancer	63671	102 (0.16)	63671	97 (0.15)	0.99 [0.75, 1.30]
	Renal cancer	63671	152 (0.24)	63671	153 (0.24)	0.95 [0.76, 1.19]
	Gastric cardia cancer	63671	12 (0.02)	63671	12 (0.02)	1.03 [0.46, 2.32]
	Thyroid cancer	63671	79 (0.12)	63671	65 (0.10)	1.16 [0.83, 1.61]
	Uterine cancer	63671	108 (0.17)	63671	108 (0.17)	0.94 [0.72, 1.23]
Tirzepatide	Composite cancer	19599	107 (0.55)	19599	138 (0.70)	0.81 [0.62, 1.05]
	Breast cancer	19599	42 (0.21)	19599	50 (0.26)	0.92 [0.60, 1.40]
	Colorectal cancer	19599	15 (0.08)	19599	19 (0.10)	0.80 [0.40, 1.61]
	Liver cancer	19599	<10 (0.05)	19599	<10 (0.05)	1.90 [0.57, 6.32]
	Multiple myeloma	19599	<10 (0.05)	19599	<10 (0.05)	0.75 [0.23, 2.46]
	Oesophageal cancer	19599	<10 (0.05)	19599	<10 (0.05)	0.52 [0.05, 5.75]
	Ovarian cancer	19599	<10 (0.05)	19599	<10 (0.05)	0.35 [0.07, 1.79]
	Pancreatic cancer	19599	15 (0.08)	19599	<10 (0.05)	1.63 [0.71, 3.76]
	Renal cancer	19599	13 (0.07)	19599	18 (0.09)	0.76 [0.37, 1.58]
	Thyroid cancer	19599	<10 (0.05)	19599	<10 (0.05)	0.38 [0.12, 1.22]
	Uterine cancer	19599	<10 (0.05)	19599	16 (0.08)	0.32 [0.11, 0.87]
Bariatric surgery	Composite cancer	9575	281 (2.93)	9575	359 (3.75)	0.82 [0.70, 0.96]
	Breast cancer	9575	96 (1.00)	9575	118 (1.23)	0.86 [0.66, 1.13]
	Colorectal cancer	9575	37 (0.39)	9575	47 (0.49)	0.82 [0.53, 1.26]
	Liver cancer	9575	14 (0.15)	9575	31 (0.32)	0.49 [0.26, 0.91]
	Multiple myeloma	9575	12 (0.13)	9575	19 (0.20)	0.69 [0.33, 1.41]
	Oesophageal cancer	9575	<10 (0.10)	9575	<10 (0.10)	0.55 [0.16, 1.89]
	Ovarian cancer	9575	16 (0.17)	9575	13 (0.14)	1.21 [0.57, 2.54]
	Pancreatic cancer	9575	21 (0.22)	9575	27 (0.28)	0.82 [0.47, 1.46]
	Renal cancer	9575	34 (0.36)	9575	31 (0.32)	1.15 [0.71, 1.87]
	Gastric cardia cancer	9575	<10 (0.10)	9575	<10 (0.10)	1.84 [0.44, 7.68]
	Thyroid cancer	9575	23 (0.24)	9575	32 (0.33)	0.76 [0.45, 1.31]
	Uterine cancer	9575	32 (0.33)	9575	62 (0.65)	0.53 [0.34, 0.81]

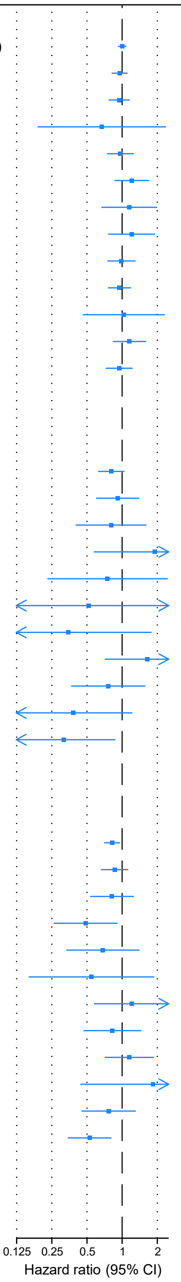

The hazard ratio of gallbladder cancer could not be calculated for tirzepatide or bariatric surgery analysis, or for gastric cardia cancer for tirzepatide analysis due to no events occurring in one or more cohorts.

DPP4: Dipeptidyl peptidase-4 inhibitor; **CI:** Confidence Interval.

The RECORD statement – **The RECORD statement – checklist of items, extended from the STROBE statement, that should be reported in observational studies using routinely collected health data.**

	**Item No.**	**STROBE items**	**Location in manuscript where items are reported**	**RECORD items**	**Location in manuscript where items are reported**
**Title and abstract**					
	1	(a) Indicate the study's design with a commonly used term in the title or the abstract (b) Provide in the abstract an informative and balanced summary of what was done and what was found	Title and abstract page 1-3	RECORD 1.1: The type of data used should be specified in the title or abstract. When possible, the name of the databases used should be included.<br><br>RECORD 1.2: If applicable, the geographic region and timeframe within which the study took place should be reported in the title or abstract.<br><br>RECORD 1.3: If linkage between databases was conducted for the study, this should be clearly stated in the title or abstract.	Title and abstract page 1-3
**Introduction**					
Background rationale	2	Explain the scientific background and rationale for the investigation being reported	Introduction page 5-7		
Objectives	3	State specific objectives, including any prespecified hypotheses	Introduction page 5-7		
**Methods**					
Study Design	4	Present key elements of study design early in the paper	Materials and Methods page 7-10		

Setting	5	Describe the setting, locations, and relevant dates, including periods of recruitment, exposure, follow-up, and data collection	Materials and Methods "Study Population" section page 8		
Participants	6	*(a) Cohort study* - Give the eligibility criteria, and the sources and methods of selection of participants. Describe methods of follow-up<br><br>*(b) Cohort study* - For matched studies, give matching criteria and number of exposed and unexposed	Materials and Methods "Study Population" section page 8	RECORD 6.1: The methods of study population selection (such as codes or algorithms used to identify subjects) should be listed in detail. If this is not possible, an explanation should be provided.<br><br>RECORD 6.2: Any validation studies of the codes or algorithms used to select the population should be referenced. If validation was conducted for this study and not published elsewhere, detailed methods and results should be provided.<br><br>RECORD 6.3: If the study involved linkage of databases, consider use of a flow diagram or other graphical display to demonstrate the data linkage process, including the number of individuals with linked data at each stage.	Materials and Methods "Data Source" and "Study Population" section page 7-8
Variables	7	Clearly define all outcomes, exposures, predictors, potential confounders, and effect modifiers. Give diagnostic criteria, if applicable.	Materials and Methods "Study Population", "Outcomes", and "Confounding factors" sections page 8-9	RECORD 7.1: A complete list of codes and algorithms used to classify exposures, outcomes, confounders, and effect modifiers should be provided. If these cannot be reported, an explanation should be provided.	Materials and Methods "Study Population", "Outcomes", and "Confounding factors" sections

					page 8-9
Data sources/ measurement	8	For each variable of interest, give sources of data and details of methods of assessment (measurement).<br>Describe comparability of assessment methods if there is more than one group	Materials and Methods "Study Population", "Outcomes", and "Confounding factors" sections page 8-9		
Bias	9	Describe any efforts to address potential sources of bias	NA		
Study size	10	Explain how the study size was arrived at	Materials and Methods "Study Population" section page 8		
Quantitative variables	11	Explain how quantitative variables were handled in the analyses. If applicable, describe which groupings were chosen, and why	Materials and Methods "Statistical analysis" section page 9-10		

Statistical methods	12	(a) Describe all statistical methods, including those used to control for confounding<br>(b) Describe any methods used to examine subgroups and interactions<br>(c) Explain how missing data were addressed<br>(d) *Cohort study* - If applicable, explain how loss to follow-up was addressed<br>(e) Describe any sensitivity analyses	Materials and Methods "Statistical analysis" section page 9-10		
Data access and cleaning methods		..		RECORD 12.1: Authors should describe the extent to which the investigators had access to the database population used to create the study population.<br><br>RECORD 12.2: Authors should provide information on the data cleaning methods used in the study.	Materials and Methods "Statistical analysis" section page 9-10
Linkage		..		RECORD 12.3: State whether the	Materials and Methods "Data Source" section

				study included person-level, institutional-level, or other data linkage across two or more databases. The methods of linkage and methods of linkage quality evaluation should be provided.	page 7
**Results**					
Participants	13	(a) Report the numbers of individuals at each stage of the study (*e.g.*, numbers potentially eligible, examined for eligibility, confirmed eligible, included in the study, completing follow-up, and analysed)<br>(b) Give reasons for non-participation at each stage.<br>(c) Consider use of a flow diagram		RECORD 13.1: Describe in detail the selection of the persons included in the study (*i.e.*, study population selection) including filtering based on data quality, data availability and linkage. The selection of included persons can be described in the text and/or by means of the study flow diagram.	
Descriptive data	14	(a) Give characteristics of study participants (*e.g.*, demographic, clinical, social) and information on exposures and potential confounders<br>(b) Indicate the number of participants with missing data for each variable of interest<br>(c) *Cohort study* - summarise follow-up time (*e.g.*, average and total amount)	Table 1		

Outcome data	15	*Cohort study* - Report numbers of outcome events or summary measures over time	Figure 1		

Main results	16	(a) Give unadjusted estimates and, if applicable, confounder-adjusted estimates and their precision (e.g., 95% confidence interval). Make clear which confounders were adjusted for and why they were included<br>(b) Report category boundaries when continuous variables were categorized<br>(c) If relevant, consider translating estimates of relative risk into absolute risk for a meaningful time period	Figure 1		
Other analyses	17	Report other analyses done—e.g., analyses of subgroups and interactions, and sensitivity analyses	NA		
**Discussion**					
Key results	18	Summarise key results with reference to study objectives	Discussion page 17-20		

Limitations	19	Discuss limitations of the study, taking into account sources of potential bias or imprecision. Discuss both direction and magnitude of any potential bias	Discussion page 28 - 29	RECORD 19.1: Discuss the implications of using data that were not created or collected to answer the specific research question(s). Include discussion of misclassification bias, unmeasured confounding, missing data, and changing eligibility over time, as they pertain to the study being reported.	
Interpretation	20	Give a cautious overall interpretation of results considering objectives, limitations, multiplicity of analyses, results from similar studies, and other relevant evidence	Discussion page 17 - 29		
Generalisability	21	Discuss the generalisability (external validity) of the study results	Discussion page 20 - 27		
**Other Information**					
Funding	22	Give the source of funding and the role of the funders for the present study and, if applicable, for the original study on which the present article is based	Acknowledgements page 30		
Accessibility of protocol, raw data, and programming code		..		RECORD 22.1: Authors should provide information on how to access any supplemental information such as the study protocol, raw data, or programming code.	Materials and Methods "Study Population", "Outcomes", and "Confounding factors" sections page 8-9

*Reference: Benchimol EI, Smeeth L, Guttmann A, Harron K, Moher D, Petersen I, Sørensen HT, von Elm E, Langan SM, the RECORD Working Committee. The REporting of studies Conducted using Observational Routinely-collected health Data (RECORD) Statement. *PLoS Medicine* 2015;

in press.

*Checklist is protected under Creative Commons Attribution (CC BY) license.

*Reference: Benchimol EI, Smeeth L, Guttmann A, Harron K, Moher D, Petersen I, Sørensen HT, von Elm E, Langan SM, the RECORD Working Committee. The REporting of studies Conducted using Observational Routinely-collected health Data (RECORD) Statement. *PLoS Medicine* 2015; in press.

*Checklist is protected under Creative Commons Attribution (CC BY) license.
